# Vascular health, diabetes, *APOE *and dementia: the Aging, Demographics, and Memory Study

**DOI:** 10.1186/alzrt43

**Published:** 2010-06-24

**Authors:** David J Llewellyn, Iain A Lang, Fiona E Matthews, Brenda L Plassman, Mary AM Rogers, Lewis B Morgenstern, Gwenith G Fisher, Mohammed U Kabeto, Kenneth M Langa

**Affiliations:** 1 Public Health and Epidemiology Group, Peninsula Medical School, Barrack Road, Exeter, EX2 5DW, UK; 2 MRC Biostatistics Unit, Institute of Public Health, Robinson Way, Cambridge, CB2 0SR, UK; 3 Program in Epidemiology of Dementia, Duke University Medical Center, 905 W Main Street, Durham, NC, 27701, USA; 4 Department of Internal Medicine, University of Michigan, 300 N. Ingalls St., Ann Arbor, MI, 48109, USA; 5 Veterans Affairs Center for Practice Management and Outcomes Research, 2215 Fuller Rd, Ann Arbor, MI, 48105, USA; 6 Department of Neurology, University of Michigan, 1500 E. Medical Center Drive Ann Arbor, MI, 48109, USA; 7 Institute for Social Research, University of Michigan, 406 Thompson St., Ann Arbor, MI, 48106, USA

## Abstract

**Introduction:**

Evidence from clinical samples and geographically limited population studies suggests that vascular health, diabetes and apolipoprotein ε4 (APOE) are associated with dementia.

**Methods:**

A population-based sample of 856 individuals aged 71 years or older from all contiguous regions of the United States received an extensive in-home clinical and neuropsychological assessment in 2001-2003. The relation of hypertension, diabetes, heart disease, stroke, medication usage, and APOE ε4 to dementia was modelled using adjusted multivariable logistic regression.

**Results:**

Treated stroke (odds ratio [OR] 3.8, 95% confidence interval [CI] 2.0, 7.2), untreated stroke (OR 3.5, 95% CI 1.7, 7.3), and APOE ε4 (OR 2.8, 95% CI 1.7, 4.5) all increased the odds of dementia. Treated hypertension was associated with lower odds of dementia (OR 0.5, 95% CI 0.3, 1.0). Diabetes and heart disease were not significantly associated with dementia. A significant interaction was observed between APOE ε4 and stroke (P = 0.001).

**Conclusions:**

Data from the first dementia study that is representative of the United States population suggest that stroke, the APOE ε4 allele and their interaction are strongly associated with dementia.

## Introduction

Identification of modifiable risk factors for Alzheimer's disease (AD), vascular dementia (VaD), and other dementias could potentially lead to a reduction in the human and economic costs these conditions place on aging populations. Better prevention of vascular disease and better treatment of vascular risk factors, in particular, may hold promise for decreasing the incidence of these debilitating disorders [1,2]. A number of related vascular risk factors and conditions -- for example, hypertension, diabetes, heart disease, and stroke -- have been studied to determine their relation to AD and VaD. However, many of these prior studies have been based on highly selected clinical samples or community-based samples from geographically localized areas, thus raising questions about the generalizability of findings [3].

Studies of blood pressure and prevalent dementia have noted an inverse relation in late-life [4], perhaps indicating that the dementia processes itself leads to lower blood pressure [5]. Incidence studies suggest that midlife hypertension is a risk factor for dementia in late-life, and that low diastolic and very high systolic pressure in late-life may also be risk factors [4,5]. Observational studies and randomized controlled trials provide limited evidence for a protective effect of antihypertensive therapy [4-9]. However, bias was introduced to previous trials due to the large proportion of placebo patients given active treatment, and the number of patients lost to follow-up with differential drop-out. The impact of hypertension on AD is controversial and remains to be clarified.

Evidence for the relation between diabetes and dementia has also been mixed. Studies of the prevalence of diabetes in those with established dementia have provided equivocal results [10], perhaps due to methodological issues such as survival bias or the effects of AD on glucose metabolism [11]. Results from incidence studies have been more consistent, suggesting that dementia is 50 to 100% more likely in individuals with diabetes, and that the association may be slightly stronger for patients with VaD than AD [10]. The clinical relevance of possible underlying pathophysiological mechanisms remains unclear, although elevated glycosylated hemoglobin (HbA1c) levels appear to increase the risk of dementia [12], and treatments improving glycemic control may therefore reduce the risk of dementia.

There are conflicting reports on the possible association between heart disease and dementia. Several studies have reported a higher risk of dementia or cognitive impairment in heart disease patients [13-16], whereas other studies suggest no association [17,18]. Further research is needed to establish whether these discrepancies reflect methodological artifacts, and to clarify the relation between heart disease and dementia with greater precision.

The prevalence of dementia in people with a history of stroke is approximately 3.4 to 5.8 times higher than in those without [19,20], and the incidence of dementia over 10 to 25 years in those with a history of stroke is about double that observed in those free of stroke [13,14]. The presence of cerebrovascular disease and a temporal relation between cerebrovascular disease and the onset of dementia are typically central diagnostic criteria for VaD [15-17]. However, cerebrovascular disease may be identified by neurological examination or brain imaging rather than a history of clinical stroke, and a considerable proportion of stroke patients do not develop dementia or VaD [18]. The association between AD and stroke remains uncertain due to a lack of conceptual and taxonomic consensus, although dementia is commonly associated with concomitant cerebrovascular and AD pathology in community-based autopsy studies [19].

Genetic predisposition may also modulate the association between vascular risk factors, diabetes, and dementia [20]. Apolipoprotein E (APOE) plays a central role in lipid metabolism, and is the only established genetic risk factor for late-onset dementia [21]. Some previous studies suggest that the association between dementia and hypertension [22], diabetes [23], heart disease [24], or stroke [25] may be particularly strong among APOE ε4 allele carriers, although other studies suggest an additive effect with no effect modification [15].

We aimed to assess the relation between vascular health, diabetes, APOE and dementia using data from the Aging, Demographics, and Memory Study (ADAMS). The ADAMS is the first population-based study of dementia in the USA to include subjects from all regions of the country, while using a single standardized diagnostic protocol in a population-based sample [3]. The ADAMS has recently been utilized to estimate the prevalence of dementia [16] and cognitive impairment without dementia [17] in the USA.

## Materials and methods

### Participants

**Figure 1:**
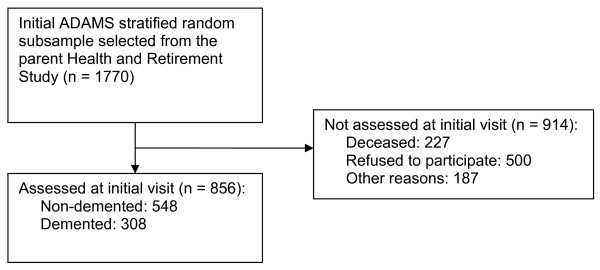
**Study flow diagram**. ADAMS, Aging, Demographics and Memory Study.

The ADAMS sample design and selection procedures are summarized below and described in detail elsewhere [3]. The ADAMS sample was drawn from the larger Health and Retirement Study (HRS), an ongoing prospective cohort study of individuals that was designed to investigate the social, health and economic implications of aging [26]. The HRS began in 1992 and was expanded in 1998 to become a representative sample of all persons in the contiguous USA older than 50 years of age. Data were collected from community living and institutionalized adults primarily through telephone interviews, with an overall response rate of 81% [27]. The ADAMS sample was derived from a stratified random subsample of 1,770 individuals aged 70 years or older from five cognitive strata based on participants' cognitive test scores or proxy-reported cognition from the most recent HRS interview (in 2000 or 2002). The three highest cognitive strata were further stratified by age (70 to 79 years vs. 80 years and older) and sex to ensure adequate numbers in each subgroup. A detailed description of the ADAMS sample, non-participants and the population weights used to ensure population representativeness has been published previously [3]. ADAMS participants did not differ significantly from nonparticipants in age, sex, or education, but were more likely to be African-American (*P *< 0.05), to have been a self-respondent at the prior HRS wave (*P *< 0.05), and to have scored in the normal range on the HRS cognitive test in the prior wave (*P *< 0.05) [3]. ADAMS initial assessments were conducted between July 2001 and December 2003, after a mean period of 13.3 months (standard deviation (SD) 6.9) from the last HRS interview. Participants were aged 71 years or older at the initial ADAMS assessment. A total of 856 individuals (56% of the nondeceased target sample) participated in all phases of the dementia assessment (Figure 1) [3].

The institutional review boards at Duke University Medical Center and the University of Michigan approved the study procedures, and all study participants or their surrogates provided informed consent.

### Measures

Full details of the ADAMS assessment and diagnostic procedures are described elsewhere [3]. A nurse and a neuropsychology technician conducted an assessment of all participants at their residence. The following information about the participant was collected from a knowledgeable informant: chronological history of cognitive symptoms, current neuropsychiatric symptoms, measures of severity of cognitive and functional impairment, and family history of memory problems. During the assessment, the participant completed a battery of neuropsychological measures, a measure of depressive symptoms, a standardized neurological examination, blood pressure measurements, collection of buccal DNA samples for APOE genotyping (presence of the ε4 allele), and a seven-minute videotaped segment covering portions of the cognitive status and neurological examinations. Medical record releases were also sought to obtain relevant neuroimaging and laboratory results from participants' physicians. A consensus expert panel of neurologists, neuropsychologists, geriatric psychiatrists, and internists reviewed all information for all participants collected during the in-home assessment first without and then with medical records and assigned final diagnoses. The consensus panel used clinical judgment to assign the final diagnosis based on currently accepted standardized international criteria for dementia (*Diagnostic and Statistical Manual of Mental Disorders *(*DSM*)-III-R and *DSM*-IV) [28,29], AD (National Institute of Neurological and Communicative Disorders and Stroke **- **Alzheimer's Disease and Related Disorders Association (NINCDS-ADRDA)) [30], and VaD (National Institute of Neurological Disorders and Stroke - Association Internationale pour la Recherché et l'Enseignement en Neurosciences (NINDS-AIREN)) [31]. The consensus panel also made an estimate of the year of dementia onset based on the available cognitive and additional data. Non-demented participants were also further divided into two categories: normal cognitive function and cognitive impairment without dementia (defined as mild cognitive or functional impairment that did not meet the criteria for dementia, or performance that was both below expectation and at least 1.5 SDs below published norms on any neuropsychological test) [17]. It should be noted that the distribution of diagnoses becomes nationally representative only after population weights are applied [3].

The presence of hypertension, diabetes, heart disease, and stroke was established based upon self- or proxy-report of these conditions at the HRS interview prior to the ADAMS assessment. During the HRS interview, each respondent was asked whether a doctor had ever told them that they had hypertension ('high blood pressure or hypertension?'), diabetes ('diabetes or high blood sugar?'), heart disease ('heart attack, coronary heart disease, angina, congestive heart failure, or other heart problems?'), or a stroke ('stroke?'). In addition to self-report of hypertension, blood pressure was directly measured during the ADAMS assessment and individuals who had systolic blood pressure of 140 mmhg or above, or diastolic blood pressure of 90 mmhg or above on two successive measurements were categorized as hypertensive. Linked Medicare claims data from 1991 were also used to confirm presence of stroke. The ADAMS subject or informant was asked to provide a list of all current medications used, and to provide the bottles for each of these medications for the nurse to confirm usage. We used these medication data to assign a 'treated' or 'untreated' status based on the current use of one or more relevant medication for each condition (hypertension, diabetes, heart disease, and stroke).

### Statistical analysis

The probability that an individual participated in ADAMS was modelled using logistic regression as a function of covariates derived from the HRS, such as age, sex, education, marital status, HRS cognition scores, residential status, and health conditions, to develop nonresponse adjustments to the sample selection weights. Population sample weights were then constructed to take into account the probabilities of selection in the stratified sample design and to adjust for differential nonparticipation [3]. Multivariable logistic regression models were then used to determine the relation of vascular health (hypertension, heart disease and stroke), diabetes and APOE to all-cause dementia and the common dementia subtypes (AD and VaD). We adjusted for age, sex, education, ethnicity, and HRS wave in basic models, and also corrected for all of the other variables in fully adjusted models. Adjustment for stroke was not incorporated in the fully adjusted VaD models and we did not examine the association between stroke and VaD because cerebrovascular disease is a central diagnostic criterion for VaD [31]. In a series of secondary analyses we examined the associations between vascular conditions, diabetes and dementia separately by treatment status and examined interactions with APOE genotype. In a sensitivity analysis we also excluded those with cognitive impairment without dementia from the non-demented control group. In a second sensitivity analysis we restricted conditions to those reported in the HRS before the estimated onset of dementia in order to help establish the temporal relation of observed associations. All analyses were conducted using Stata SE version 9.2 (StataCorp, College Station, TX, USA).

## Results

The characteristics of the ADAMS population are shown in Table 1. Just over half of the sample was comprised of women, over half had received at least nine years of education, and almost three-quarters were white in ethnic origin. Over half of the study population had hypertension and had heart disease, and stroke and diabetes were also relatively common. More than one-quarter of the sample were APOE ε4 carriers. Participants with dementia were generally older, less educated and with a higher proportion of women than non-demented controls. The prevalence of diabetes was lower in the demented group, whereas the prevalence of stroke and APOE ε4 was higher.

**Table 1:** Baseline characteristics of the study population

Variables	Non-demented controls (n = 548)	All-cause dementia (n = 308)	Alzheimer's disease (n = 229)	Vascular dementia (n = 48)
Mean age (SD), years	78.3 (6.3)	84.5 (6.9)	85.5 (6.7)	84.0 (6.2)
Women, n (%)	288 (59.4)	213 (68.5)	170 (71.5)	28 (62.1)
**Education, n (%)**				
8 years or less	166 (14.8)	125 (33.5)	93 (32.3)	18 (33.8)
9 years or more	382 (85.2)	183 (66.5)	136 (67.7)	30 (66.2)
**Ethnic origin, n (%)**				
White	395 (87.7)	218 (83.4)	162 (82.1)	36 (87.3)
Black	92 (6.9)	67 (12.4)	49 (12.9)	9 (10.5)
Hispanic	61 (5.4)	23 (4.2)	18 (5.0)	3 (2.2)
HRS wave*, n (%)				
2000	157 (31.8)	136 (34.4)	98 (34.5)	26 (42.3)
2002	391 (68.2)	172 (65.6)	131 (65.6)	22 (57.7)
Mean MMSE total (SD)	27.0 (4.1)	15.9 (6.4)	15.4 (6.4)	18.7 (5.9)
**Hypertension, n (%)**				
Untreated	98 (14.9)	61 (23.8)	53 (27.0)	5 (24.0)
Treated	308 (58.6)	150 (44.2)	100 (38.8)	32 (56.8)
**Diabetes, n (%)**				
Untreated	14 (2.1)	16 (4.7)	12 (4.2)	1 (1.3)
Treated	109 (17.9)	31 (11.9)	12 (7.2)	9 (12.5)
**Heart disease, n (%)**				
Untreated	36 (6.1)	32 (11.5)	25 (9.9)	3 (18.3)
Treated	144 (22.3)	79 (28.2)	53 (28.2)	18 (31.8)
**Stroke, n (%)**				
Untreated	114 (17.7)	150 (51.6)	98 (46.0)	36 (82.4)
Treated	22 (3.8)	24 (7.6)	13 (5.2)	10 (15.2)
APOE ε4, n (%)	125 (24.4)	110 (38.6)	87 (38.4)	11 (35.7)

Means and percentages are weighted to adjust for the sampling design. *Participants were selected from the 2000 and 2002 waves of the Health and Retirement Study (HRS).. APOE ε4, apolipoprotein ε4; MMSE, Mini-Mental State Examination (scores range from 0 to 30 where higher scores indicate better cognitive function); SD, standard deviation.

The associations observed between vascular health, diabetes, APOE and dementia are shown in Table 2. We first adjusted for age, sex, education, ethnicity and HRS wave (basic adjusted models), and then added all other covariates (fully adjusted models). Stroke and APOE ε4 were clearly associated with dementia, and these results changed little by additional adjustment. Stroke and APOE ε4 were also associated with increased odds of AD. There was little suggestion of an association between hypertension, diabetes or heart disease and AD. APOE ε4 was also associated with VaD, and the strength of the association between APOE and the main dementia subtypes was similar. The associations between hypertension, diabetes and heart disease and VaD were not statistically significant.

**Table 2:** Logistic regression models illustrating the odds of all-cause dementia, Alzheimer's disease and vascular dementia (95% confidence intervals) by vascular conditions, diabetes and APOE genotype

	All-cause dementia		Alzheimer's disease		Vascular dementia	
	Basic adjusted models*	Fully adjusted models†	Basic adjusted models*	Fully adjusted models†	Basic adjusted models*	Fully adjusted models‡
Hypertension	0.7 (0.4-1.2)	0.7 (0.4-1.2)	0.6 (0.4-1.1)	0.7 (0.4-1.2)	1.7 (0.6-5.0)	2.0 (0.6-6.3)
Diabetes	0.9 (0.5-1.7)	1.1 (0.5-2.1)	0.6 (0.3-1.6)	0.7 (0.3-1.7)	0.8 (0.4-1.4)	0.5 (0.2-1.1)
Heart disease	1.5 (0.9-2.6)	1.4 (0.8-2.4)	1.4 (0.8-2.6)	1.5 (0.8-2.9)	2.2 (0.7-7.6)	2.6 (0.8-8.4)
Stroke	3.7 (2.2-6.2)	3.7 (2.0-6.9)	2.4 (1.5-3.9)	2.3 (1.3-4.0)	§	§
APOE ε4	2.5 (1.7-3.9)	2.8 (1.7-4.5)	2.6 (1.5-4.6)	2.6 (1.5-4.8)	2.3 (1.0-5.2)	2.3 (1.1-5.1)

Population weights are used to adjust for the sampling design. *Adjusted for age, sex, education, ethnicity, and Health and Retirement Study (HRS) wave. †Adjusted for age, sex, education, ethnicity, HRS wave, and all other variables in the table. ‡Adjusted for age, sex, education, ethnicity, HRS wave, and all other variables in the table excluding stroke. §Not examined as stroke is a central diagnostic criterion for vascular dementia [31]. APOE ε4, apolipoprotein ε4.

Table 3 shows the association between untreated and treated vascular conditions, diabetes and dementia. Treated hypertension was associated with lower odds of dementia and lower odds of AD. There was some suggestion that untreated heart disease might increase the odds of dementia (*P *= 0.07). Treated and untreated stroke were associated with higher odds of dementia and to a lesser extent AD.

**Table 3:** Logistic regression models illustrating the odds of all-cause dementia, Alzheimer's disease and vascular dementia (95% confidence intervals) by untreated and treated vascular conditions and diabetes

	All-cause dementia		Alzheimer's disease		Vascular dementia	
	Basic adjusted models*	Fully adjusted models†	Basic adjusted models*	Fully adjusted models†	Basic adjusted models*	Fully adjusted models‡
**Hypertension**						
Untreated	1.3 (0.6-2.9)	1.3 (0.6-3.0)	1.4 (0.7-2.8)	1.5 (0.7-3.1)	2.6 (0.5-14.3)	2.9 (0.4-19.9)
Treated	0.6 (0.3-1.0)	0.5 (0.3-1.0)	0.4 (0.2-0.8)	0.5 (0.2-1.0)	1.4 (0.5-4.5)	1.7 (0.5-5.3)
**Diabetes**						
Untreated	2.5 (0.7-8.1)	2.3 (0.7-7.2)	2.1 (0.6-7.0)	2.1 (0.6-6.8)	0.8 (0.1-8.6)	(Insufficient cases observed)
Treated	0.7 (0.4-1.5)	0.9 (0.5-1.8)	0.4 (0.1-1.5)	0.5 (0.1-1.6)	0.8 (0.4-1.4)	0.6 (0.3-1.2)
**Heart disease**						
Untreated	2.1 (1.4-3.3)	1.8 (1.0-3.3)	1.7 (0.8-3.7)	1.6 (0.5-4.6)	4.2 (0.8-20.8)	4.1 (0.9-18.8)
Treated	1.3 (0.7-2.5)	1.2 (0.6-2.4)	1.3 (0.6-2.8)	1.5 (0.7-3.1)	1.8 (0.6-5.5)	2.1 (0.7-6.6)
**Stroke**						
Untreated	3.8 (2.2-6.6)	3.8 (2.0-7.2)	2.6 (1.6-4.2)	2.4 (1.3-4.2)	§	§
Treated	3.2 (1.6-6.4)	3.5 (1.7-7.3)	1.8 (0.9-3.7)	1.9 (0.8-4.2)	§	§

Population weights are used to adjust for the sampling design. 'Untreated' and 'Treated' categories were defined on the basis of a nurse inspection of medicine bottles currently used. *Adjusted for age, sex, education, ethnicity, and Health and Retirement Study (HRS) wave. †Adjusted for age, sex, education, ethnicity, HRS wave, apolipoprotein ε4 (APOE ε4) status, and all other variables in the table. ‡Adjusted for age, sex, education, ethnicity, HRS wave, APOE ε4 status, and all other variables in the table excluding stroke. §Not examined as stroke is a central diagnostic criterion for vascular dementia [31].

There was little suggestion of an interaction between hypertension, diabetes, or heart disease and APOE ε4 in fully adjusted models (*P *> 0.1). However, there was a significant interaction between stroke and APOE ε4. We therefore stratified the association between stroke and dementia by APOE ε4 genotype (Table 4). There was little difference between the basic and fully adjusted models. Those with both stroke and the APOE ε4 allele were approximately 15 times more likely to be demented.

**Table 4:** Logistic regression model illustrating the odds of all-cause dementia (95% confidence intervals) by combinations of stroke and APOE genotype

	n	Basic adjusted models*	Fully adjusted models†
Stroke - APOE ε4 -	388	Reference	Reference
Stroke + APOE ε4 -	219	2.2 (1.2-4.0)	2.2 (1.1-4.2)
Stroke - APOE ε4 +	151	1.4 (0.8-2.4)	1.3 (0.7-2.5)
Stroke + APOE ε4 +	84	14.8 (6.3-34.9)	14.9 (5.8-38.0)
*P *for stroke × APOE ε4 interaction = 0.001†			

Population weights are used to adjust for the sampling design. *Adjusted for age, sex, education, ethnicity, and Health and Retirement Study (HRS) wave. †Adjusted for age, sex, education, ethnicity, HRS wave, heart disease, hypertension, and diabetes. APOE ε4, apolipoprotein ε4.

Excluding participants who were cognitively impaired without dementia (n = 241) from the non-demented control group gave the same pattern of results (data not shown). The effect sizes increased slightly for all conditions as we were comparing demented participants with a more selective group, and confidence intervals were therefore generally increased due to the reduced sample size. Restricting vascular conditions and diabetes to conditions reported before the estimated onset of dementia gave a highly similar pattern of results. For example, stroke was more likely (odds ratio (OR) 4.8, 95% confidence interval (CI) 2.8 to 8.0) and hypertension was less likely (OR 0.4, 95% CI 0.2 to 0.6) before the onset of dementia, whereas no association was observed for diabetes or heart disease (*P *> 0.3). The interaction between stroke and APOE ε4 remained significant (*P *= 0.018).

## Discussion

In this study we investigated the associations between hypertension, diabetes, heart disease, stroke, APOE ε4 and dementia. Stroke and the APOE ε4 allele emerged as being strongly associated with dementia in the USA population. Treated hypertension was associated with decreased odds of dementia, whereas untreated heart disease emerged as being possibly associated. Treated or untreated diabetes did not appear to increase the odds of dementia. There was little evidence for an interaction between APOE ε4 and hypertension, diabetes or heart disease. However, APOE ε4 did modify the relation between stroke and dementia as those with stroke who were APOE ε4 negative had two times higher odds of dementia, while those with stroke who were APOE ε4 positive had 15 times higher odds of dementia.

Many prior studies of dementia have been based on highly selected clinical samples or community-based samples from geographically localized areas [3]. This analysis is the first to our knowledge to examine the relation between vascular conditions, diabetes and dementia in a large heterogeneous population-based sample that is representative of the contiguous USA. We incorporated an extensive in-home clinical and neuropsychological assessment to determine a consensus dementia diagnosis using a single standardized diagnostic protocol for all participants, and adjusted for a range of potential confounders.

A number of possible sources of bias should also be discussed. A proportion of the HRS sample died or were lost to follow-up before they could be assessed in the ADAMS. The response rate for the ADAMS study was also moderate (56% of the nondeceased target sample), which could pose a threat to the representativeness of the study. However, extensive information about individuals who refused to participate was available from the parent HRS, including level of cognitive function, which was incorporated in the population weights used to ensure the representativeness of the ADAMS sample. As we studied the relation between vascular conditions, diabetes and dementia cross-sectionally, bias could nevertheless have occurred due to survival effects. The association with the conditions would have been inflated if mortality in those with vascular conditions or diabetes was lower in those that were also demented. More likely, the combination of vascular conditions, diabetes and dementia may have led to increased mortality and an underestimation of the relation. However, the same pattern of results was observed when we restricted our analyses to conditions reported before the estimated onset of dementia. Although dementia was diagnosed by generally accepted international criteria and a central consensus committee, the possibility of misdiagnosis must be noted, particularly when distinguishing between Alzheimer's disease, vascular dementia and other dementias.

Although previous studies provide equivocal evidence to suggest that antihypertensives may be neuroprotective [4-9], the prevalence of dementia and AD was lower in treated hypertensives than untreated hypertensives in the present study. Thus the importance of hypertension may be underestimated if studies fail to account for antihypertensive treatment. Our results add to the inconsistent literature to suggest that diabetes [10] and heart disease [32-37] may have a limited association with dementia on a population level. Our findings are consistent with previous studies suggesting a three to six times higher prevalence of dementia in stroke patients [19,20]. Interestingly, the prevalence of stroke was higher in participants with AD, and the association between stroke and dementia was not limited to all-cause dementia or VaD. Our results are also in line with studies demonstrating that APOE ε4 is an important risk factor for dementia [21].

Hypertension is a risk factor for stroke, ischemic white matter lesions, silent infarcts, and general atherosclerosis, and has also been related to AD pathology [5]. Dementia is commonly associated with concomitant cerebrovascular and AD pathology in community-based autopsy studies [19]. Possible mechanisms linking stroke and dementia include vascular lesions, white-matter changes, or combinations of these and AD pathology [18]. APOE ε4 is associated with hippocampal, amygdala, and entorhinal cortex atrophy, increased brain atrophy, increased white matter hyperintensity volumes and altered cerebral blood flow and glucose metabolism [38], however the mechanisms for these changes remain unclear. Although some studies suggest that the association between vascular conditions and dementia may be modified by APOE ε4 [22-25], we only observed a significant interaction with stroke. Based on these findings, we postulate that stroke and APOE may lead to dementia via related mechanisms.

## Conclusions

Data from the first dementia study that is representative of the USA population suggest that antihypertensive medications may be neuroprotective, whereas stroke and APOE ε4 are strongly associated with dementia. It remains to be established which pathological mechanisms are clinically important on a population level, although our evolving understanding of the etiology of dementia has the potential to inform more effective interventions and treatments. Interventions designed to reduce the incidence of stroke may also be effective in reducing the incidence of dementia, particularly in APOE ε4 carriers.

## Abbreviations

AD: alzheimer's disease; ADAMS: Aging: Demographics: and Memory Study; APOE ε4: apolipoprotein E epsilon 4; CI: confidence interval; DSM: Diagnostic and Statistical Manual of Mental Disorders; HbA1c: glycosylated hemoglobin; HRS: Health and Retirement Study; NINCDS-ADRDA: National Institute of Neurological and Communicative Disorders and Stroke **- **Alzheimer's Disease and Related Disorders Association; NINDS-AIREN: National Institute of Neurological Disorders and Stroke - Association Internationale pour la Recherché et l'Enseignement en Neurosciences; OR: odds ratio; SD: standard deviation; VaD: vascular dementia.

## Competing interests

The authors declare that they have no competing interests.

## Authors' contributions

DJL and KML conceived of the study. DJL, BLP, MAMR, GGF, MUK and KML acquired the data. DJL, IAL, FEM, MUK and KML contributed to the analytic approach and performed the statistical analysis. DJL, IAL and KML drafted the manuscript. All authors contributed to the interpretation of findings and the revision of the manuscript for important intellectual content.

## References

[B1] Gorelick PB Risk factors for vascular dementia and Alzheimer disease Stroke 2004 35 2620 2622 10.1161/01.STR.0000143318.70292.47 15375299

[B2] Kuller LH Lopez OL Jagust WJ Becker JT DeKosky ST Lyketsos C Kawas C Breitner JC Fitzpatrick A Dulberg C Determinants of vascular dementia in the Cardiovascular Health Cognition Study Neurology 2005 64 1548 1552 10.1212/01.WNL.0000160115.55756.DE 15883315 PMC3378359

[B3] Langa KM Plassman BL Wallace RB Herzog AR Heeringa SG Ofstedal MB Burke JR Fisher GG Fultz NH Hurd MD The Aging, Demographics, and Memory Study: study design and methods Neuroepidemiology 2005 25 181 191 10.1159/000087448 16103729

[B4] Qiu C Winblad B Fratiglioni L The age-dependent relation of blood pressure to cognitive function and dementia Lancet Neurol 2005 4 487 499 10.1016/S1474-4422(05)70141-1 16033691

[B5] Skoog I Gustafson D Update on hypertension and Alzheimer's disease Neurol Res 2006 28 605 611 10.1179/016164106X130506 16945211

[B6] Lithell H Hansson L Skoog I Elmfeldt D Hofman A Olofsson B Trenkwalder P Zanchetti A The Study on Cognition and Prognosis in the Elderly (SCOPE): principal results of a randomized double-blind intervention trial J Hypertens 2003 21 875 886 10.1097/00004872-200305000-00011 12714861

[B7] Di Bari M Pahor M Franse LV Shorr RI Wan JY Ferrucci L Somes GW Applegate WB Dementia and disability outcomes in large hypertension trials: lessons learned from the systolic hypertension in the elderly program (SHEP) trial Am J Epidemiol 2001 153 72 78 10.1093/aje/153.1.72 11159149

[B8] Forette F Seux ML Staessen JA Thijs L Babarskiene MR Babeanu S Bossini A Fagard R Gil-Extremera B Laks T The prevention of dementia with antihypertensive treatment: new evidence from the Systolic Hypertension in Europe (Syst-Eur) study Arch Intern Med 2002 162 2046 2052 10.1001/archinte.162.18.2046 12374512

[B9] Forette F Seux ML Staessen JA Thijs L Birkenhager WH Babarskiene MR Babeanu S Bossini A Gil-Extremera B Girerd X Laks T Lilov E Moisseyev V Tuomilehto J Vanhanen H Webster J Yodfat Y Fagard R Prevention of dementia in randomised double-blind placebo-controlled Systolic Hypertension in Europe (Syst-Eur) trial Lancet 1998 352 1347 1351 10.1016/S0140-6736(98)03086-4 9802273

[B10] Biessels GJ Staekenborg S Brunner E Brayne C Scheltens P Risk of dementia in diabetes mellitus: a systematic review Lancet Neurol 2006 5 64 74 10.1016/S1474-4422(05)70284-2 16361024

[B11] Watson GS Craft S Modulation of memory by insulin and glucose: neuropsychological observations in Alzheimer's disease Eur J Pharmacol 2004 490 97 113 10.1016/j.ejphar.2004.02.048 15094077

[B12] Gao L Matthews FE Sargeant LA Brayne C An investigation of the population impact of variation in HbA1c levels in older people in England and Wales: from a population based multi-centre longitudinal study BMC Public Health 2008 8 54 10.1186/1471-2458-8-54 18267013 PMC2275259

[B13] Kokmen E Whisnant JP O'Fallon WM Chu CP Beard CM Dementia after ischemic stroke: a population-based study in Rochester, Minnesota (1960-1984) Neurology 1996 46 154 159 8559366 10.1212/wnl.46.1.154

[B14] Ivan CS Seshadri S Beiser A Au R Kase CS Kelly-Hayes M Wolf PA Dementia after stroke: the Framingham Study Stroke 2004 35 1264 1268 10.1161/01.STR.0000127810.92616.78 15118167

[B15] Jin YP Ostbye T Feightner JW Di Legge S Hachinski V Joint effect of stroke and APOE 4 on dementia risk: the Canadian Study of Health and Aging Neurology 2008 70 9 16 10.1212/01.wnl.0000284609.77385.03 17978275

[B16] Plassman BL Langa KM Fisher GG Heeringa SG Weir DR Ofstedal MB Burke JR Hurd MD Potter GG Rodgers WL Steffens DC Willis RJ Wallace RB Prevalence of dementia in the United States: the aging, demographics, and memory study Neuroepidemiology 2007 29 125 132 10.1159/000109998 17975326 PMC2705925

[B17] Plassman BL Langa KM Fisher GG Heeringa SG Weir DR Ofstedal MB Burke JR Hurd MD Potter GG Rodgers WL Steffens DC McArdle JJ Willis RJ Wallace RB Prevalence of cognitive impairment without dementia in the United States Ann Intern Med 2008 148 427 434 18347351 10.7326/0003-4819-148-6-200803180-00005 PMC2670458

[B18] Leys D Henon H Mackowiak-Cordoliani MA Pasquier F Poststroke dementia Lancet Neurol 2005 4 752 759 10.1016/S1474-4422(05)70221-0 16239182

[B19] (CFAS) NGotMRCCFaASM Pathological correlates of late-onset dementia in a multicentre, community-based population in England and Wales Lancet 2001 357 169 175 10.1016/S0140-6736(00)03589-3 11213093

[B20] Haan MN Shemanski L Jagust WJ Manolio TA Kuller L The role of APOE epsilon4 in modulating effects of other risk factors for cognitive decline in elderly persons Jama 1999 282 40 46 10.1001/jama.282.1.40 10404910

[B21] Ertekin-Taner N Genetics of Alzheimer's disease: a centennial review Neurol Clin 2007 25 611 667 v 10.1016/j.ncl.2007.03.009 17659183 PMC2735049

[B22] Qiu C Winblad B Fastbom J Fratiglioni L Combined effects of APOE genotype, blood pressure, and antihypertensive drug use on incident AD Neurology 2003 61 655 660 12963757 10.1212/wnl.61.5.655

[B23] Peila R Rodriguez BL Launer LJ Type 2 diabetes, APOE gene, and the risk for dementia and related pathologies: The Honolulu-Asia Aging Study Diabetes 2002 51 1256 1262 10.2337/diabetes.51.4.1256 11916953

[B24] Hofman A Ott A Breteler MM Bots ML Slooter AJ van Harskamp F van Duijn CN Van Broeckhoven C Grobbee DE Atherosclerosis, apolipoprotein E, and prevalence of dementia and Alzheimer's disease in the Rotterdam Study Lancet 1997 349 151 154 10.1016/S0140-6736(96)09328-2 9111537

[B25] Slooter AJ Tang MX van Duijn CM Stern Y Ott A Bell K Breteler MM Van Broeckhoven C Tatemichi TK Tycko B Hofman A Mayeux R Apolipoprotein E epsilon4 and the risk of dementia with stroke. A population-based investigation Jama 1997 277 818 821 10.1001/jama.277.10.818 9052712

[B26] Juster FT Suzman R An Overview of the Health and Retirement Study The Journal of Human Resources 1995 30 7 56 10.2307/146277

[B27] Sample Sizes and Response Rates http://hrsonline.isr.umich.edu/intro/sho_uinfo.php?hfyle=sample_new_v3&xtyp=2

[B28] Association AP Diagnostic and statistical manual of mental disorders 1987 3 Washington, DC: American Psychiatric Assoc

[B29] Association AP Diagnostic and Statistical Manual of Mental Disorders 1994 4 Washington, DC: American Psychiatric Assoc

[B30] McKhann G Drachman D Folstein M Katzman R Price D Stadlan EM Clinical diagnosis of Alzheimer's disease: report of the NINCDS-ADRDA Work Group under the auspices of Department of Health and Human Services Task Force on Alzheimer's Disease Neurology 1984 34 939 944 6610841 10.1212/wnl.34.7.939

[B31] Roman GC Tatemichi TK Erkinjuntti T Cummings JL Masdeu JC Garcia JH Amaducci L Orgogozo JM Brun A Hofman A Moody DM O'Brien MD Yamaguchi T Grafman J Drayer BP Bennett DA Fisher M Ogata J Kokmen E Bermejo F Wolf PA Gorelick PB Bick KL Pajeau AK Bell MA DeCarli C Culebras A Korczyn AD Bogousslavsky J Hartmann A Scheinberg P Vascular dementia: diagnostic criteria for research studies. Report of the NINDS-AIREN International Workshop Neurology 1993 43 250 260 8094895 10.1212/wnl.43.2.250

[B32] Breteler MM Claus JJ Grobbee DE Hofman A Cardiovascular disease and distribution of cognitive function in elderly people: the Rotterdam Study Bmj 1994 308 1604 1608 8025427 10.1136/bmj.308.6944.1604 PMC2540432

[B33] Aronson MK Ooi WL Morgenstern H Hafner A Masur D Crystal H Frishman WH Fisher D Katzman R Women, myocardial infarction, and dementia in the very old Neurology 1990 40 1102 1106 2356012 10.1212/wnl.40.7.1102

[B34] Qiu C Winblad B Marengoni A Klarin I Fastbom J Fratiglioni L Heart failure and risk of dementia and Alzheimer disease: a population-based cohort study Arch Intern Med 2006 166 1003 1008 10.1001/archinte.166.9.1003 16682574

[B35] Lindsay J Hebert R Rockwood K The Canadian Study of Health and Aging: risk factors for vascular dementia Stroke 1997 28 526 530 9056606 10.1161/01.str.28.3.526

[B36] Bursi F Rocca WA Killian JM Weston SA Knopman DS Jacobsen SJ Roger VL Heart disease and dementia: a population-based study Am J Epidemiol 2006 163 135 141 10.1093/aje/kwj025 16293716

[B37] Petrovitch H White L Masaki KH Ross GW Abbott RD Rodriguez BL Lu G Burchfiel CM Blanchette PL Curb JD Influence of myocardial infarction, coronary artery bypass surgery, and stroke on cognitive impairment in late life Am J Cardiol 1998 81 1017 1021 10.1016/S0002-9149(98)00082-4 9576163

[B38] Cherbuin N Leach LS Christensen H Anstey KJ Neuroimaging and APOE genotype: a systematic qualitative review Dement Geriatr Cogn Disord 2007 24 348 362 10.1159/000109150 17911980

